# A Systematic Review and Meta-Analysis of Animal Studies Testing Intra-Arterial Chilled Infusates After Ischemic Stroke

**DOI:** 10.3389/fneur.2020.588479

**Published:** 2021-01-06

**Authors:** Lane J. Liddle, Christine A. Dirks, Brittany A. Fedor, Mohammed Almekhlafi, Frederick Colbourne

**Affiliations:** ^1^Department of Psychology, University of Alberta, Edmonton, AB, Canada; ^2^Neuroscience and Mental Health Institute, University of Alberta, Edmonton, AB, Canada; ^3^Calgary Stroke Program, Calgary, AB, Canada

**Keywords:** therapeutic hypothermia (TH), meta-analysis, intraarterial cooling, neuroprotection, focal ischemia, MCAO (middle cerebral artery occlusion)

## Abstract

**Background:** As not all ischemic stroke patients benefit from currently available treatments, there is considerable need for neuroprotective co-therapies. Therapeutic hypothermia is one such co-therapy, but numerous issues have hampered its clinical use (e.g., pneumonia risk with whole-body cooling). Some problems may be avoided with brain-specific methods, such as intra-arterial selective cooling infusion (IA-SCI) into the arteries supplying the ischemic tissue.

**Objective:** Our research question was about the efficacy of IA-SCI in animal middle cerebral artery occlusion models. We hypothesized that IA-SCI would be beneficial, but translationally-relevant study elements may be missing (e.g., aged animals).

**Methods:** We completed a systematic review of the PubMed database following the PRISMA guidelines on May 21, 2020 for animal studies that administered IA-SCI in the peri-reperfusion period and assessed infarct volume, behavior (primary meta-analytic endpoints), edema, or blood-brain barrier injury (secondary endpoints). Our search terms included: “focal ischemia” and related terms, “IA-SCI” and related terms, and “animal” and related terms. Nineteen studies met inclusion criteria. We adapted a methodological quality scale from 0 to 12 for experimental design assessment (e.g., use of blinding/randomization, *a priori* sample size calculations).

**Results:** Studies were relatively homogenous (e.g., all studies used young, healthy animals). Some experimental design elements, such as blinding, were common whereas others, such as sample size calculations, were infrequent (median methodological quality score: 5; range: 2–7). Our analyses revealed that IA-SCI provides benefit on all endpoints (mean normalized infarct volume reduction = 23.67%; 95% CI: 19.21–28.12; mean normalized behavioral improvement = 35.56%; 95% CI: 25.91–45.20; mean standardized edema reduction = 0.95; 95% CI: 0.56–1.34). Unfortunately, blood-brain barrier assessments were uncommon and could not be analyzed. However, there was substantial statistical heterogeneity and relatively few studies. Therefore, exploration of heterogeneity *via* meta-regression using saline infusion parameters, study quality, and ischemic duration was inconclusive.

**Conclusion:** Despite convincing evidence of benefit in ischemic stroke models, additional studies are required to determine the scope of benefit, especially when considering additional elements (e.g., dosing characteristics). As there is interest in using this treatment alongside current ischemic stroke therapies, more relevant animal studies will be critical to inform patient studies.

## Introduction

Ischemic stroke is one of the leading global causes of death and disability (1). Acute therapies, namely, tissue plasminogen activator (tPA) and mechanical thrombectomy (MT) are the only clinically-proven therapies for acute ischemic stroke (2–4). As a thrombolytic agent, tPA can reduce neurological deficits and improve functional outcomes when given to patients shortly after ictus [within ~4.5 h; (2, 3, 5)]. Similarly, clot retrieval *via* MT devices can improve patient outcomes within 6–24 h of symptom onset, depending on patient condition (6, 7). Unfortunately, not all patients that receive these treatments become functionally independent (8). For example, in some cases, <50% of the tPA-treated patient population may have successful recanalization after ischemic stroke (2). In other cases, patients do not benefit despite successful recanalization for several reasons [e.g., older age, delayed presentation, poor collateral status; (9–11)]. Without additional treatment options or co-therapies, disability rates in such patients will remain unchanged.

Therapeutic hypothermia (TH) is a long-studied approach for treating ischemic injury (12, 13). Indeed, TH provides demonstrable benefit in cardiac arrest and hypoxic-ischemic encephalopathy (14, 15). However, clinical application of TH in ischemic stroke has proven to be difficult, with multiple clinical trials prematurely ended owing to safety and feasibility concerns (16, 17). Thus, the limitations of current cooling techniques must be addressed. Until recently, TH methods have been limited to systemic cooling by use of cooling blankets and endovascular approaches, or *via* exogenous regional cooling through cooling helmets or nasopharyngeal approaches (3, 18). Systemic cooling often takes hours to reach target temperatures and frequently results in complications [e.g., shivering, pneumonia; (3, 19, 20)]. Despite avoiding systemic complications, exogenous regional cooling may not penetrate subcortical brain regions that are commonly affected after ischemic stroke, and prolonged cooling with these methods is not yet feasible (3). Therefore, although efficacious in similar brain injuries, the use of TH in ischemic stroke will remain limited without advancements in TH methodologies.

Intra-arterial selective cooling infusion [IA-SCI; (21)] is a newer TH method that directly targets the injured parenchyma by infusing cool (i.e., <35°C) substances into the arteries supplying the infarcted region (22, 23). The infusate may be physiologically inert (e.g., saline, Ringer's solution) or active (e.g., magnesium sulfate), and may be a useful co-therapy to current endovascular approaches. Current studies commonly infuse saline into the common carotid artery or internal carotid artery following middle cerebral artery occlusion (MCAO) with a hollow microcatheter or intraluminal filament (24, 25). With the feasibility and efficacy of IA-SCI shown in multiple preclinical studies, recent pilot trials have shown that IA-SCI is feasible in focal ischemia patients (21, 26). Accordingly, IA-SCI may avoid complications observed with other TH techniques, as localizing TH effects limits systemic complications and allows for faster temperature reductions (19). In addition to the benefits of brief hypothermia [e.g., decreased cellular metabolism, blood-brain barrier improvement (22, 27)], IA-SCI studies have shown that flushing the ischemic vasculature improves cerebral blood flow, clears the vasculature of inflammatory mediators, and reduces oxidative stress (25, 28, 29). Many of these mechanisms may provide benefit as a supplemental therapy for endovascular therapies, particularly for those that do not benefit from endovascular therapies alone (21). In sum, IA-SCI is a pluripotent treatment that may be an eligible co-therapy for patients who may not benefit from current therapies.

In order to reduce the substantial disability rates associated with ischemic stroke, current preclinical research aims to generate and test neuroprotective treatments that are clinically relevant to reduce disease burden and improve patient outcomes (30, 31). Initially, exploratory studies are conducted to identify target mechanisms and establish safety and efficacy of putative neuroprotectants, and later confirmatory elements are added (32, 33). In the early stages, research is often conducted in young, healthy, male animals for cost and simplicity reasons. Later studies are expected to introduce clinically-relevant and more rigorous experimental design elements [e.g., all features of good laboratory practice, comparison of sex differences, use of aged animals, use of animals with comorbidities; (34–36)]. Translational rigor is critical to consider as some studies have shown that experiments failing to consider these elements result in biased outcome assessments [i.e., inflated effect sizes; (37, 38)]. Moreover, high-quality experimental reporting is critical to reproducibility and ethical, cost-effective use of laboratory animals (39, 40). Altogether, effective translation of putative neuroprotectants is thought to be predicated on high quality experimental design and reporting.

While the feasibility and efficacy of the IA-SCI protocol has been examined in animal studies (18), no meta-analysis exists with a focus on IA-SCI. Thus, through systematic review and meta-analysis, we quantified the efficacy of IA-SCI, analyzed the translational quality of IA-SCI studies, and highlighted future opportunities. Our efficacy assessment is based on histological and behavioral outcomes, as recommended by the STAIR and RIGOR publications regarding translational rigor in stroke neuroprotection research (41, 42). As TH commonly reduces blood-brain barrier disruption and edema, secondary analysis centered on blood-brain barrier improvement and edema reduction, which are both key issues, especially in severe strokes. Experimental design parameters (e.g., randomization, blinding) were qualitatively assessed and combined into a methodological quality scale (MQS) for study quality assessment. Altogether, this study provides an updated and focused review on the use of IA-SCI as a supplemental therapy to current endovascular approaches for ischemic stroke and the efficacy of IA-SCI administered at the time of reperfusion.

## Methods

### Search Terms and Inclusion Criteria

Although we did not pre-register this study, we upheld PRISMA standards (meeting all other aspects of the PRISMA checklist) during our systematic search of the PubMed database from Canada for all published English-language literature on May 21, 2020 using the following terms: (focal ischemia OR stroke OR ischemic stroke OR MCAO OR middle cerebral artery occlusion) AND (intra-arterial saline OR saline infusion OR IACS OR SI-AC OR ICSI OR carotid infusion OR LCI OR local cooling infusion OR LEVI OR local endovascular infusion) AND (hypothermia OR local cooling OR focal hypothermia OR focal cooling OR cooling OR therapy OR therapeutic hypothermia OR stroke therapy OR efficacy OR translation) AND (rat OR mouse OR gerbil OR rodent OR primate OR monkey OR dog OR cat OR lamb OR patient OR human) NOT [coronary (Text Word) OR myocardial (Text Word)]. All studies that were identified during the systematic review were included in the meta-analysis.

For our inclusion criteria we followed the PICOS framework using English-language only studies (43). Our population of interest was any animal study of MCAO. Our inclusion criteria for the intervention included any study using IA-SCI within 1 h before, during, or after reperfusion. Our inclusion criteria for the comparator group was the following hierarchy: sham catheter insertion but no infusion, sham procedure without catheter insertion, pure MCAO without infusion or sham procedure, carotid catheterization with homeothermic (37°C) saline infusion. We selected this hierarchy because some studies have discovered histological and/or behavioral benefit with normothermic carotid saline infusion and thus this procedure may not be an adequate “null effect” control (25, 44). Our inclusion criteria for outcomes were infarct volume and/or behavior and/or edema and/or blood-brain barrier assessment. If histology or behavior was assessed at multiple time points, only the comparison at the latest time point was extracted [long-term outcomes are recommended by the STAIR guidelines; (41)]. In terms of study design, we accepted prospective studies with concurrent control groups. Studies that did not meet the above criteria were excluded. Article screening and data extraction were completed by 2 reviewers using Covidence software, as recommended by the Cochrane Handbook for Systematic Reviews of Interventions and others (45–47). Outcome data were extracted into a shared spreadsheet by one reviewer and all data were validated by a second reviewer. In general, we collected descriptive statistics, experimental parameters, and specific treatment parameters for any study that assessed infarct volume, behavior, edema, or the blood-brain barrier (see Supplementary Material for all variables extracted). All ordinal behavioral rating scales were converted to represent low scores indicating less deficit by subtracting the mean value by the value of the maximum score (e.g., Garcia behavioral assessment scale). Finally, one study reported no behavioral benefit using IA-SCI, but the data were not presented and could not be obtained from the authors (no response). Therefore, we assumed a conservative effect size estimate of Cohen's d = 0 and an average variability estimate.

### Study Quality Assessment

We adapted previously used study quality scales to analyze study quality and possible bias risk. The elements of our scale are informed by guidelines and publications for translationally-rigorous neuroprotection research [e.g., STAIR and RIGOR; (38, 39, 41, 48)]. For study quality assessment, we considered: study pre-registration, exclusion statement, inclusion of both sexes, aged and/or comorbid animals, *a priori* sample size calculations, randomization, blinding to outcomes and/or treatment, control of body temperature, clear description of infusion parameters (e.g., rate and/or duration, temperature), and conflict of interest statements. The characteristics of interest were ranked by most prevalent to least prevalent to give a snapshot of the current literature characteristics, and directions for future research (Table 1). In Table 2, we broke down the use of each characteristic at the level of the individual study.

**Table 1:** Methodological quality scale reporting elements for preclinical IA-SCI research in ischemic stroke and % adherence.

**Element**	**% Adherence**
Control of body temperature	100
Blinding to outcome(s)	89
Complete reporting of infusion parameters	89
Group randomization	63
Exclusion statement	58
Conflict of interest statement	47
Blinding to ischemia/IA-SCI	21
Use of male and female animals	5
Use of aged animals	0
Use of animals with comorbid conditions	0
Sample size calculation	0
A priori study registration	0

**Table 2:** Statistical summary comparison and total % of reported MQS elements (✓ = yes, χ = no).

**References**	**Pre-registration**	**Exclusion statement**	**Both sexes**	**Old age**	**Sample size calculation**	**Randomization**	**Comorbidities**	**Outcome(s) blinding**	**Ischemia/IA-SCI blinding**	**Control body temp**.	**Infusion parameters**	**Conflict of interest**	**Total score (max. 12)**
Chen et al. (44)	χ	χ	χ	χ	χ	✓	χ	✓	✓	✓	χ	✓	5
Chen et al. (27)	χ	✓	χ	χ	χ	✓	χ	✓	χ	✓	✓	✓	6
Corey et al. (49)	χ	χ	✓	χ	χ	✓	χ	✓	χ	✓	✓	χ	5
Ding et al. (23)	χ	✓	χ	χ	χ	χ	χ	✓	χ	✓	✓	χ	4
Ding et al. (25)	χ	χ	χ	χ	χ	χ	χ	✓	χ	✓	✓	χ	3
Ding et al. (22)	χ	χ	χ	χ	χ	χ	χ	χ	χ	✓	✓	χ	2
Duan et al. (50)	χ	✓	χ	χ	χ	✓	χ	✓	✓	✓	✓	✓	7
Ji et al. (51)	χ	✓	χ	χ	χ	✓	χ	✓	χ	✓	✓	✓	6
Ji et al. (28)	χ	✓	χ	χ	χ	✓	χ	✓	χ	✓	✓	χ	5
Kim et al. (52)	χ	χ	χ	χ	χ	χ	χ	✓	χ	✓	✓	χ	3
Kurisu et al. (53)	χ	✓	χ	χ	χ	χ	χ	✓	χ	✓	✓	✓	5
Kurisu et al. (54)	χ	✓	χ	χ	χ	✓	χ	✓	χ	✓	✓	✓	6
Li et al. (55)	χ	✓	χ	χ	χ	χ	χ	✓	χ	✓	✓	χ	4
Song et al. (24)	χ	χ	χ	χ	χ	✓	χ	✓	χ	✓	χ	χ	3
Wang et al. (56)	χ	χ	χ	χ	χ	✓	χ	✓	χ	✓	✓	✓	5
Wei et al. (57)	χ	✓	χ	χ	χ	✓	χ	✓	✓	✓	✓	✓	7
Wu et al. (58)	χ	χ	χ	χ	χ	✓	χ	χ	χ	✓	✓	χ	3
Wu et al. (59)	χ	✓	χ	χ	χ	✓	χ	✓	✓	✓	✓	✓	7
Zhao et al. (60)	χ	✓	χ	χ	χ	χ	χ	✓	χ	✓	✓	χ	4
Total (%)	0	58	5	0	0	63	0	89	21	100	89	47	

### Statistical Analysis

Data were analyzed using R statistical software (version 3.5.1, Vienna, Austria). Because of the varying MCAO durations and infusion parameters, we chose to use random-effects meta-analysis models for all endpoints with the Dersimonian-Laird estimator. In cases when one control group serviced multiple treatment groups, the sample size of the control group was divided by the number of treatment groups serviced [described in (61)]. Whenever possible, we normalized our data to sham or baseline values [recommended and used in (61, 62)]. The normalization procedure yielded normalized mean difference effect sizes that were expressed as a percentage of sham or baseline performance, and our data were converted in this way only if sham or baseline data were available or could be reasonably assumed (61). In our analysis, we considered the latest infarct volume measurement for each study. We statistically combined the effect sizes from the latest assessment of each behavioral test within each study by weighing the outcome from each behavioral task using the inverse variance weighting method (61). There was little variability in the timing of edema measurement across studies. However, varied edema measurement methodologies (i.e., tissue wet-dry weight or histological measurement) meant that we had to use standardized mean differences as our effect size measure rather than normalized effect sizes. Hedge's G [advantageous in the context of relatively small sample sizes; (61)] was used when standardized mean differences were used. Meta-regression was used to investigate heterogeneity, and MCAO duration, saline infusion parameters (temperature, rate, volume), and methodological quality were parameters in the meta-regression model. Planned sensitivity analyses were conducted if unexpected or extreme results were obtained in the forest plots.

## Results

### Search Results

**Figure 1:**
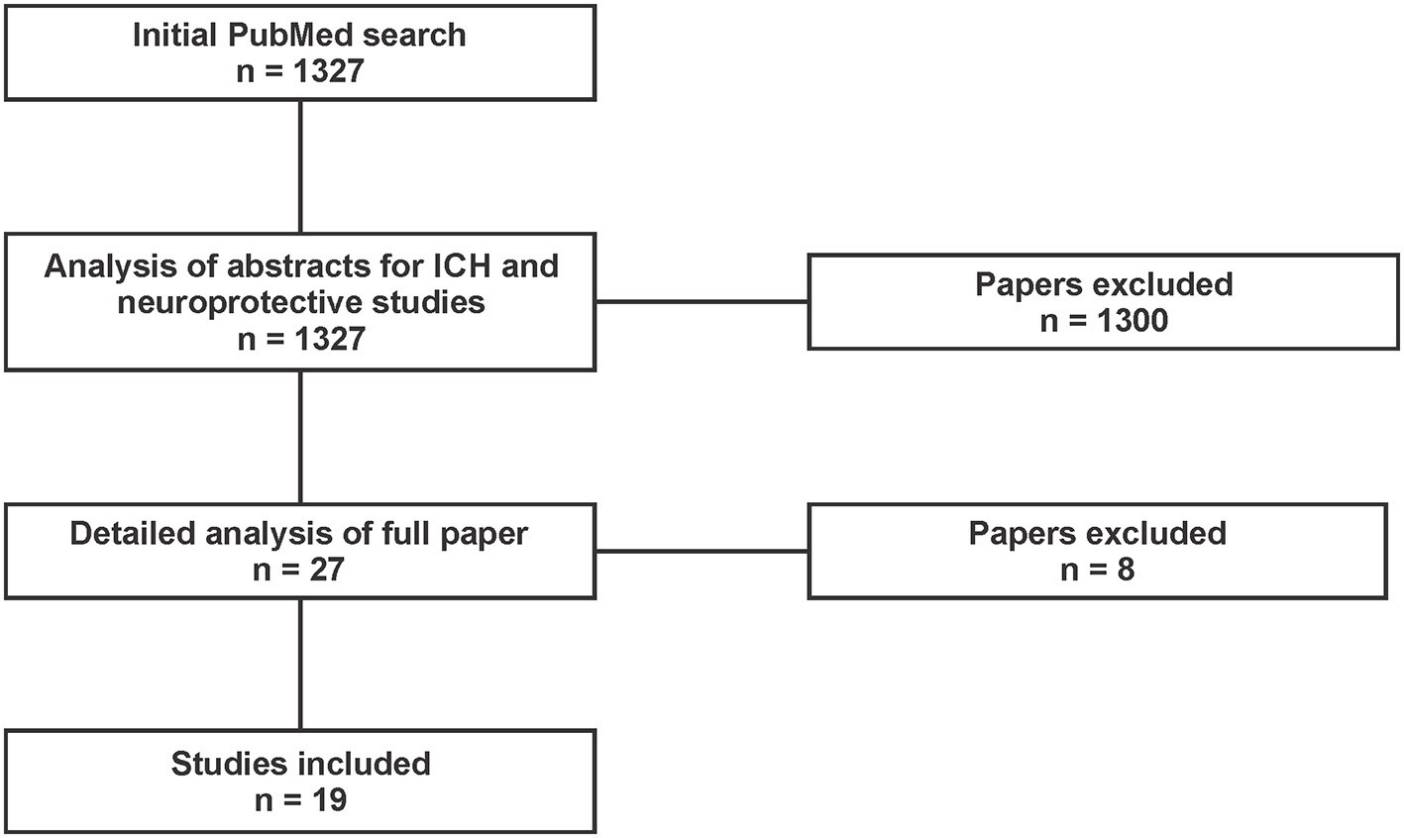
PRISMA diagram of studies returned by our PubMed search conducted on May 21, 2020. Inclusion criteria limited studies to those in animal models of intracarotid cooling after ischemic stroke that assessed infarct volume and/or behavior and/or edema (English only). Our search included the following terms: (focal ischemia OR stroke OR ischemic stroke OR MCAO OR middle cerebral artery occlusion) AND (intra-arterial saline OR saline infusion OR IACS OR SI-AC OR ICSI OR carotid infusion OR LCI OR local cooling infusion OR LEVI OR local endovascular infusion) AND (hypothermia OR local cooling OR focal hypothermia OR focal cooling OR cooling OR therapy OR therapeutic hypothermia OR stroke therapy OR efficacy OR translation) AND (rat OR mouse OR gerbil OR rodent OR primate OR monkey OR dog OR cat OR lamb OR patient OR human) NOT [coronary (Text Word) OR myocardial (Text Word)].

Of the 1,327 studies returned for title and abstract screening, 1,300 were omitted for irrelevance. Following full-text screening of the remaining 27 studies, we excluded 3 for incorrect study design, 2 for incorrect comparator, 2 for incorrect outcomes, and 1 for non-English language. This left 19 studies for data analysis and extraction (Figure 1).

### Study Characteristics

**Figure 2:**
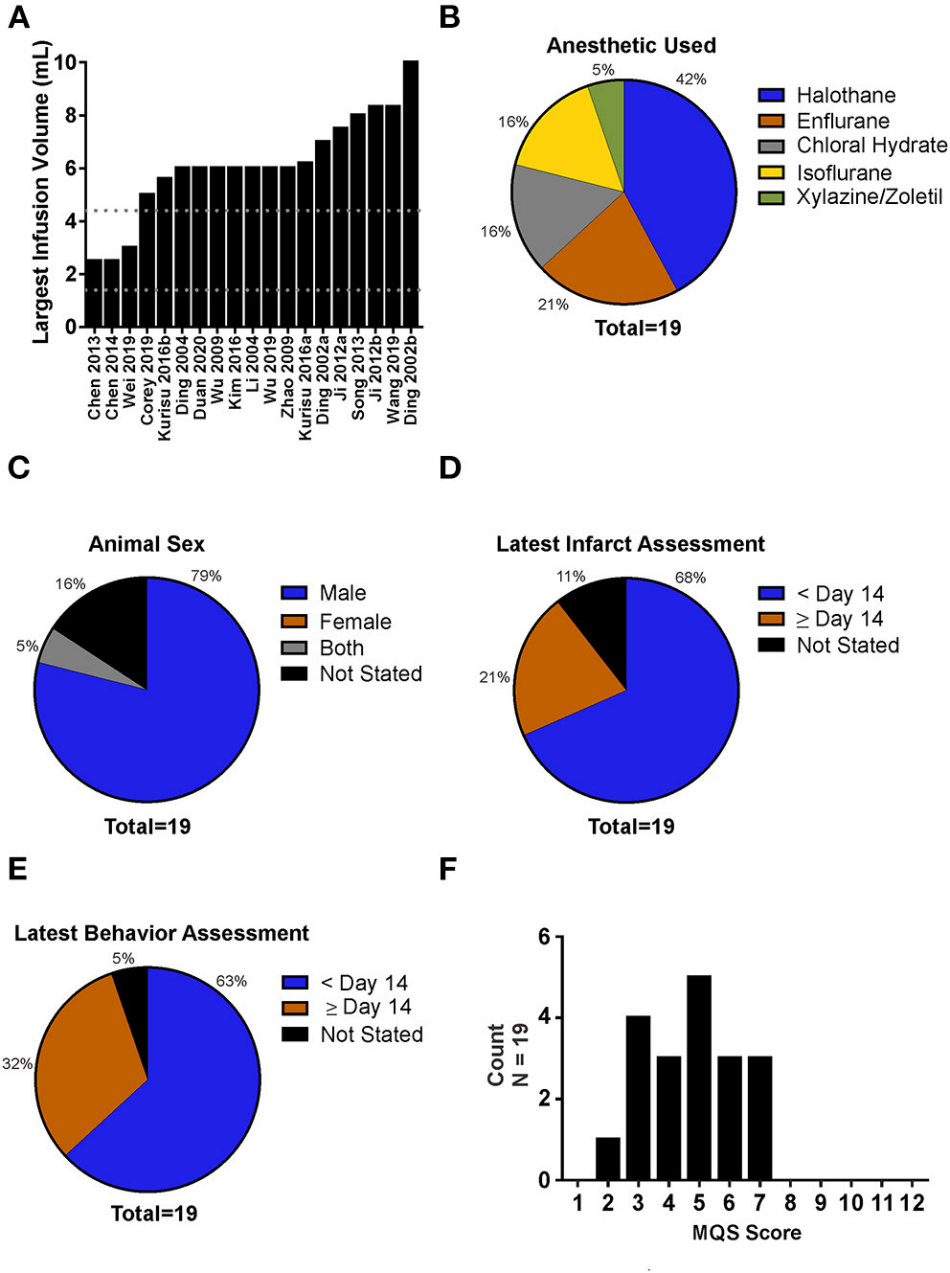
Analysis of experimental characteristics used in intra-arterial selective cooling infusion (IA-SCI) literature. **(A)** Distribution of saline infusion volumes used in IA-SCI studies. Upper and lower dotted lines represent our calculated limits for saline infusion volumes. Calculations are based on 1 L of saline being infused into a patient and scaled to brain mass (1.4 mL; lower line) and body mass (4.4 mL; upper line). **(B)** Breakdown of anesthetics used in IA-SCI neuroprotection studies. **(C)** Animal sexes used in IA-SCI neuroprotection literature. No studies used exclusively female animals. **(D)** Analysis of infarct assessment timing. A majority of studies investigated infarct volume between 0 and 14 days post-MCAO (median = 2 days post-MCAO). **(E)** Analysis of behavioral assessment timing. Similar to infarct volume assessment, a majority of studies assessed behavior between 0 and 14 days post-MCAO (median = 2 days post-MCAO). **(F)** Evaluation of methodological quality score for IA-SCI studies (max score = 12). MQS, Methodological Quality Score.

A modified PE-50 microcatheter occlusion model of MCAO (25) was the most commonly used ischemia model (58% of studies), with the classic Longa (63) suture occlusion model being used in fewer studies (37%). One study (5%) permanently occluded the MCA *via* craniotomy. Sixty-three percent of studies reported randomization, 58% exclusions, 47% conflict of interest statement, 26% complete blinding (i.e., during IA-SCI infusion and outcome assessment), and 11% stated the mortality rate (Tables 1, 2). No studies were pre-registered, used rats with comorbidities, or conducted *a priori* group size calculations. Studies infused a median of 6 mL of saline (range 2.5–10 mL; Figure 2A). The most common anesthetic used was halothane (42%) (Figure 2B). All studies used young, male Sprague-Dawley rats, except for one study which included females, but did not consider biological sex as a variable or detail the distribution of females per group (Figure 2C). All studies regulated body temperature during surgery. Eighty-four percent of studies measured the impact of IA-SCI on brain temperature. One study used multiple ischemia lengths. All studies used saline in the IA-SCI infusion.

### Tissue and Behavioral Endpoints

Out of the 19 studies analyzed, 17 assessed infarct volume, 17 assessed behavioral outcomes, and 9 assessed edema. The latest date of infarct volume and behavioral assessment was 28 days (median = 2; Figures 2D,E). Only 4 studies analyzed blood-brain-barrier integrity.

### Analysis of Methodological Quality

For an overview of our methodological quality scale and analysis of studies making use of the experimental elements included in our methodological quality scale, see Tables 1, 2. Overall, the median methodological quality score was 5, indicating low to moderate study quality, with a range from 2 to 7 (12 points maximum; Figure 2F).

### Characteristics of IA-SCI

The median IA-SCI temperature was 10°C and ranged from 0 to 23°C. Infusion volume characteristics are listed in Study Characteristics section. All studies administered the IA-SCI treatment immediately following MCAO (i.e., immediately after the occluding filament was removed), with the exception of one study that administered IA-SCI 1 h after bilateral carotid occlusion and distal occlusion of the MCAO with surgical clips *via* craniotomy. In studies that used the modified PE-50 microcatheter as an occlusion device, IA-SCI was administered immediately before reperfusion (i.e., monofilament withdrawn 1–2 mm following MCAO and IA-SCI occurred), whereas in studies using the Longa suture occlusion method, IA-SCI occurred post-reperfusion, following removal of the occluding filament, and advancement of the IA-SCI catheter. Also, in studies that used the modified PE-50 microcatheter, the infusion took place closer to the infarct (~1–2 mm from MCA origin). In studies that used the Longa suture method, infusions were closer to the internal carotid artery origin (within 5 mm of the carotid bifurcation).

### Impact of IA-SCI on Body Temperature

All 19 studies measured body temperature intraoperatively, and maintained body temperature between 36.5 and 37.5°C. Despite this, only 10 studies remarked on, tabulated, or graphically depicted the change in body temperature during and after the IA-SCI infusion. Studies reported an average nadir body temperature reduction of 0.28°C from baseline, within 5 min of IA-SCI. Thus, the average minimum body temperature achieved by IA-SCI was ~36.72°C during the infusion, with most studies indicating small, non-significant body temperature changes from baseline (range: 36.09–37.5°C). Body temperature quickly returned to baseline values after IA-SCI within 10 min, however one study reported reduced body temperature for up to 50 min.

### Impact of IA-SCI on Brain Temperature

Sixteen out of 19 studies measured brain temperature. Fourteen out of 16 studies measured brain temperature using implantable needle thermistor probes, and 2 studies did not specify their brain temperature measurement method. None performed or mentioned contralateral brain temperature measurement, an important internal control. Thirteen out of the 16 studies measured ipsilesional brain temperature in more than 1 region of interest (i.e., striatum and cortex). Studies reported an average nadir brain temperature reduction of 4°C from baseline, within 3–5 min of IA-SCI start. Thus, the average minimum brain temperature achieved by IA-SCI was ~33°C during the infusion (range: 30.5–34.8°C). Brain temperature decreases were similar in the cortex and striatum. At the cessation of IA-SCI, brain temperature often quickly returned to baseline values within 5–10 min, however some studies reported reduced brain temperatures for longer than 10 min, up to 50 min.

### Infarct Volume Results

**Figure 3:**
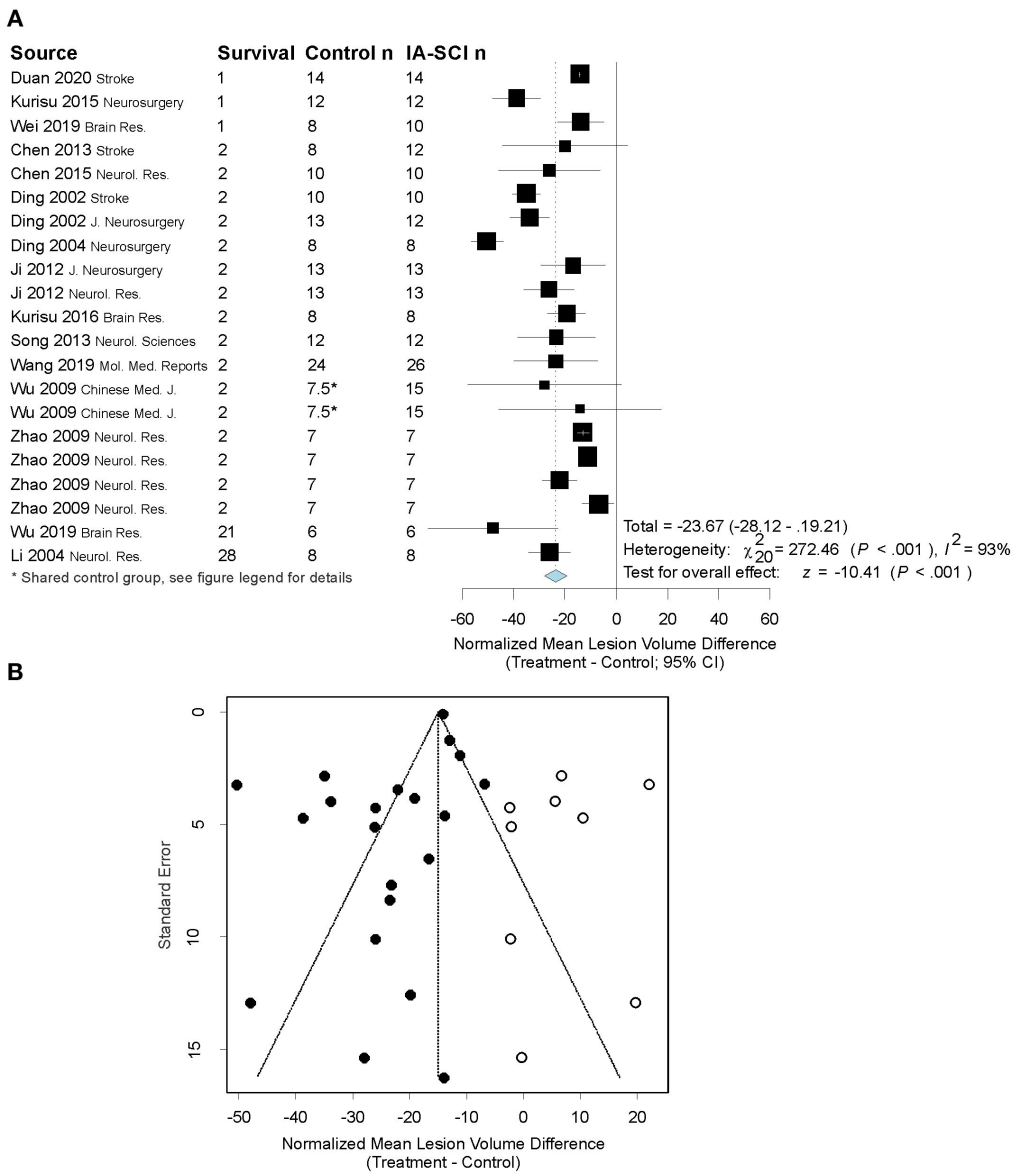
Quantitative analysis of studies that assessed infarct volume using random-effects meta-analysis. **(A)** Forest plot of studies investigating infarct volume (normalized mean difference ± 95% CI). Effect size estimates were heterogeneous, likely owing to study design differences. *Control group in (58) serviced 2 treatment groups, and thus to avoid outcome dependence, we divided the control group in half [procedure described in (61)]. **(B)** Funnel plot with trim-and-fill analysis to assess publication bias. Closed circles represent actual studies whereas open circles represent results of the trim-and-fill analysis. These results suggest that null and negative studies may be missing from the distribution (i.e., the left side of the funnel is more filled out, favoring beneficial effects of the treatment on infarct volume). Note that the effect size in Panel B is smaller than in Panel A, as this has been corrected for in the trim-and-fill analysis.

Seventeen studies assessed infarct volume; of these, 16 measured infarct volume as a “% infarction” using the contralesional hemisphere as control tissue. The remaining study analyzed infarct volume as an absolute value in cubic millimeters. Descriptive characteristics of each infarct volume assessment can be found in Table 3, with all data available in the Supplementary Material. The results of our analysis can be found in Figure 3A. In brief, our random-effects model revealed that IA-SCI significantly reduced infarct volume compared to controls [*p* < 0.0001; normalized mean reduction: 23.66 (95% CI: 19.21, 28.12)]. However, there was substantial heterogeneity among studies (I^2^ = 93%; *P* < 0.0001). Additionally, the funnel plot, Egger regression analysis, and trim-and fill bias analyses revealed that the current literature may be missing null or negative data, as the trim-and-fill analysis primarily filled the right side of the plot (Egger regression *p* = 0.01; Figure 3B). More specifically, after the trim-and-fill analysis, the normalized mean difference was 14.94%, dropping almost 10%. In an attempt to explain some heterogeneity and investigate the impact of various model parameters, we performed meta-regression using MCAO duration, saline infusion parameters, and MQS as predictors. We did not find effects of MCAO duration (*p* = 0.42), saline temperature (*p* = 0.59), saline volume (*p* = 1.0), saline rate (*p* = 0.26), nor MQS (*p* = 0.88) on infarct volume reduction.

**Table 3:** Descriptive characteristics of studies that assessed infarct volume.

**References**	**Latest assessment (*d*)**	**Control (*n*)**	**IA-SCI (*n*)**	**Normalized effect size**	**95% confidence interval**
Duan et al. (50)	1	14	14	−14.2	−14.36, −14.04
Kurisu et al. (53)	1	12	12	−38.8	−48.04, −29.56
Wei et al. (57)	1	8	10	−13.9	−22.94, −4.86
Chen et al. (44)	2	8	12	−19.9	−44.51, 4.71
Chen et al. (27)	2	10	10	−26.1	−45.9, −6.3
Ding et al. (25)	2	10	10	−35	−40.54, −29.46
Ding et al. (22)	2	13	12	−33.9	−41.7, −26.1
Ding et al. (23)	2	8	8	−50.4	−56.71, −44.09
Ji et al. (51)	2	13	13	−16.7	−29.48, −3.92
Ji et al. (28)	2	13	13	−26.2	−36.21, −16.19
Kurisu et al. (54)	2	8	8	−19.26	−26.77, −11.75
Song et al. (24)	2	12	12	−23.32	−38.4, −8.24
Wang et al. (56)	2	24	26	−23.54	−39.93, −7.15
Wu et al. (58)	2	7.5 (shared)	15	−28	−58.11, 2.11
Wu et al. (58)	2	7.5 (shared)	15	−14	−45.85, 17.85
Zhao et al. (60)	2	7	7	−13.01	−15.48, −10.54
Zhao et al. (60)	2	7	7	−11.2	−14.98, −7.42
Zhao et al. (60)	2	7	7	−22.12	−28.89, −15.35
Zhao et al. (60)	2	7	7	−6.87	−13.16, −0.58
Wu et al. (59)	21	6	6	−48	−73.3, −22.7
Li et al. (55)	28	8	8	−26	−34.32, −17.68
**Summary statistic**	**Median** **=** **2**	**Σ** **=** **210**	**Σ** **=** **232**	${\bar{\text{X}}}_{\text{Weighted}}$ **=** **−23.67**	${\bar{\text{X}}}_{\text{Weighted}}$ **=** **−28.12**, **−19.21**

### Behavioral Results

**Figure 4:**
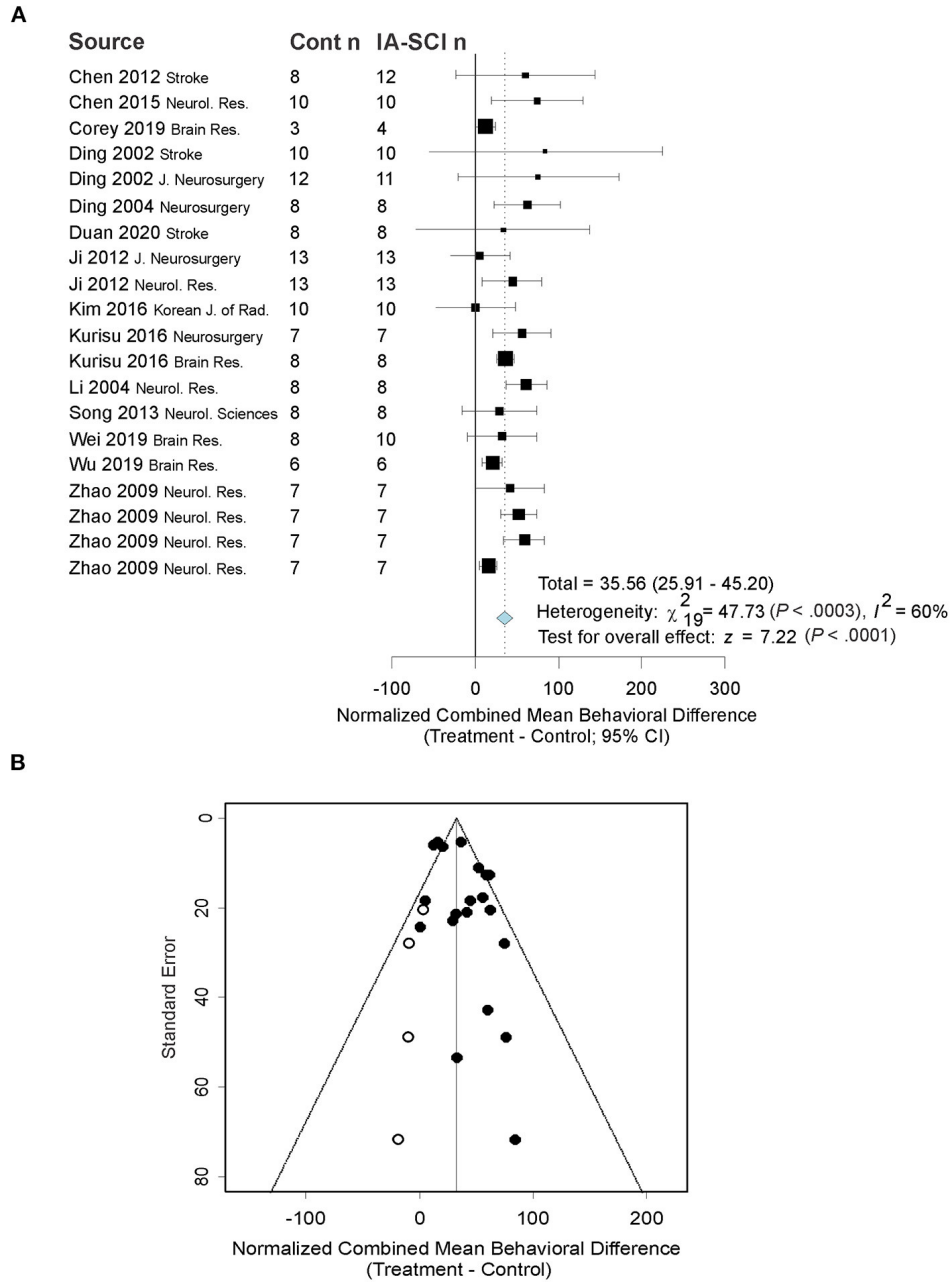
Quantitative analysis of studies that assessed behavioral outcomes using random-effects meta-analysis. **(A)** Forest plot of studies investigating behavioral outcomes (normalized mean difference ± 95% CI). Effect size estimates were very heterogeneous, likely owing to differences in MCAO duration, treatment parameters, and timing of assessment. Note: as behavioral results were combined across a number of tests, the test date could not be presented in the figure because behavioral tasks often had differing timing. **(B)** Funnel plot with trim-and-fill analysis to assess publication bias. Closed circles represent actual studies whereas open circles represent results of the trim-and-fill analysis. These results suggest that null and negative studies may be missing from the distribution (i.e., the right side of the funnel is more filled out, favoring beneficial effects of the treatment on behavioral outcomes). As in Figure 3, the effect size in Panel B is smaller than in Panel A, because of trim-and-fill correction.

Seventeen studies assessed behavior using a variety of behavioral tests. The most commonly used behavioral assessment methods were neurological deficit rating scales. Overall, studies used 1–5 behavioral tasks (median = 1), with a grand total of 41 tasks used across all studies. Data were analyzed and expressed as normalized mean differences if baseline/sham data were available or could be assumed. Two tests from one study did not provide baseline/sham data and were thus excluded. If studies used multiple tasks, their data were combined into a cumulative normalized behavioral score using a method described by Vesterinen et al. (61). Descriptive characteristics of the studies that conducted behavioral assessments can be found in Table 4, and more specific information regarding behavioral tests used (and their timing) can be found in the Supplementary Material document. Our random-effects model revealed that chilled saline infusion significantly improved behavioral outcomes compared to controls [*p* < 0.0001; normalized mean difference: 35.56 (95% CI: 25.91–45.20); Figure 4A]. However, there was substantial heterogeneity among studies (I^2^ = 60%, *p* < 0.0001). Trim-and-fill analysis and Egger regression revealed that some studies on the left side of the distribution may be missing from the literature, though the result from the Egger regression was only a trend toward significance (Egger regression *p* = 0.06; Figure 4B). However, the trim-and-fill model suggested only a small (3%) change in the normalized mean difference would occur if the filled studies were included. The meta-regression model did not find any predictive value of MCAO duration (*p* = 0.59), saline infusion parameters (*p* ≥ 0.27), or methodological quality (*p* = 0.81) on behavioral benefit after IA-SCI.

**Table 4:** Descriptive characteristics of studies that assessed behavior.

**References**	**Control (*n*)**	**IA-SCI (*n*)**	**Normalized effect size**	**95% confidence interval**	**# of tests combined**
Chen et al. (44)	8	12	59.74	−24.04, 143.52	5
Chen et al. (27)	10	10	74.58	19.81, 129.35	1
Corey et al. (49)	3	4	11.95	0.23, 23.67	1
Ding et al. (25)	10	10	83.84	−56.66, 224.34	1
Ding et al. (22)	12	11	75.52	−20.26, 171.3	4
Ding et al. (23)	8	8	61.76	21.84, 101.68	4
Duan et al. (50)	8	8	32.89	−71.49, 137.27	4
Ji et al. (51)	13	13	4.97	−31.09, 41.03	1
Ji et al. (28)	13	13	44.33	8.51, 80.16	1
Kim et al. (52)	10	10	0	−47.47, 47.47	1
Kurisu et al. (53)	7	7	55.56	20.87, 90.24	1
Kurisu et al. (54)	8	8	35.97	25.76, 46.18	1
Li et al. (55)	8	8	60.83	36.22, 85.45	5
Song et al. (24)	8	8	28.71	−16.06, 73.47	1
Wei et al. (57)	8	10	32.04	−9.63, 73.72	4
Wu et al. (59)	6	6	20.16	7.82, 32.49	2
Zhao et al. (60)	7	7	41.4	0.28, 82.51	1
Zhao et al. (60)	7	7	51.64	29.92, 73.36	1
Zhao et al. (60)	7	7	58.22	33.54, 82.9	1
Zhao et al. (60)	7	7	15.69	5.2, 26.18	1
**Summary statistic**	**Σ** **=** **147**	**Σ** **=** **153**	${\bar{\text{X}}}_{\text{Weighted}}$ **=** **35.56**	${\bar{\text{X}}}_{\text{Weighted}}$ **=** **25.91, 26.18**	**Σ** **=** **41**

### Edema Data

**Figure 5:**
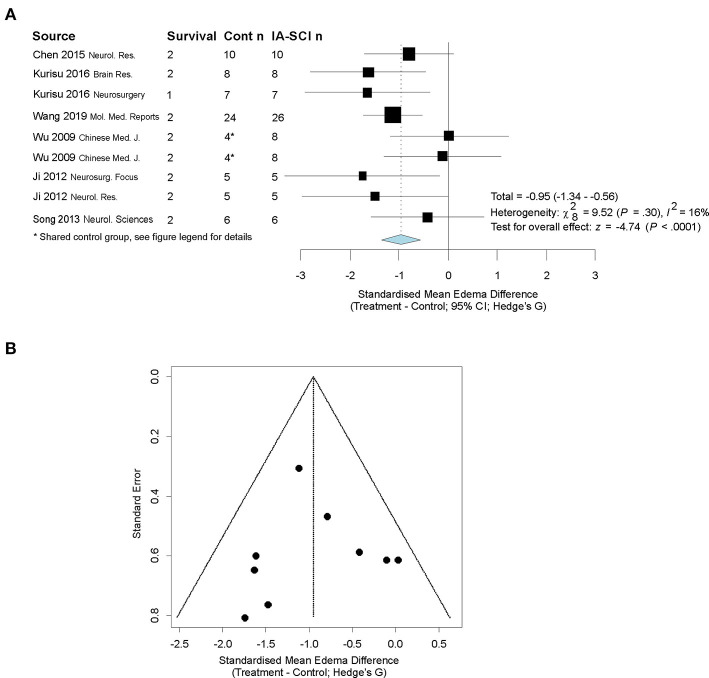
Quantitative analysis of studies that assessed edema using random-effects meta analysis. Random-effects meta-analysis was conducted because of the variability in MCAO durations and treatment infusion parameters. **(A)** Forest plot of studies investigating behavioral outcomes (Hedge's G standardized mean difference ± 95% CI). One study was not included in the final analysis owing to the exceptionally large effect size observed therein, which caused a high amount of heterogeneity in the meta-analytic model and statistical leverage in the meta-regression model. With the exceptional study removed, study heterogeneity was reduced substantially and the meta-regression model no longer showed signs of statistical leverage. *Control group in (58) serviced 2 treatment groups, and thus to avoid outcome dependence, we divided the control group in half [procedure described in (61)]. Cont *n* = control group sample size; IA-SCI *n* = IA-SCI group sample size. **(B)** Funnel plot with trim-and-fill analysis. There did not appear to be publication bias for studies using this endpoint in our analysis.

Nine studies assessed edema: 5 used the tissue wet-dry weight method, and 4 used histological methods. Our observed outcome was converted to the standardized mean difference, rather than the normalized, due to the absence of sham data in some studies and discrepancies between measurement techniques (61). Descriptive characteristics of studies that assessed edema can be found in Table 5. Our model revealed a significant effect of chilled saline infusion on edema (*p* = 0.04). However, the meta-analytic data were relatively heterogeneous (I^2^ = 85%; *p* < 0.0001). Upon inspection of the data, there was one extreme outlier (miniscule variability estimate leading to extremely high standardized effect size estimate; Hedge's G = 57) that appeared to affect heterogeneity in the model and meta-regression model when sensitivity analysis was conducted. With the outlier removed, the model still revealed a significant effect of IA-SCI on edema (*p* < 0.0001), however, our random effects model no longer returned statistical heterogeneity (I^2^ = 16%; *p* = 0.30). Additionally, our analysis of bias did not reveal remarkable publication bias by investigating funnel plots, and using Egger regression (*p* = 0.98) or trim-and-fill methods (Figure 5B). Finally, our meta-regression did not reveal a significant impact of MCAO duration (*p* = 0.91), saline infusion volume (0.82), saline temperature (*p* = 0.96), nor methodological quality (*p* = 0.49) on edema reduction. Due to limited degrees of freedom, saline rate could not be considered in the model fitting.

**Table 5:** Descriptive characteristics of studies that assessed edema.

**References**	**Latest assessment (d)**	**Control (*n*)**	**IA-SCI (*n*)**	**Standardized effect size (Hedge's G)**	**95% confidence interval**	**Edema method**
Chen et al. (27)	2	10	10	−0.79	−1.71, 0.13	Histology
Kurisu et al. (54)	2	8	8	−1.62	−2.79, −0.45	Histology
Kurisu et al. (53)	1	7	7	−1.64	−2.9, −0.37	Histology
Wang et al. (56)	2	24	26	−1.12	−1.72, −0.52	Histology
Wu et al.(58)	2	4 (shared)	8	0.03	−1.17, 1.23	Tissue wet-dry weight
Wu et al. (58)	2	4 (shared)	8	−0.11	−1.31, 1.09	Tissue wet-dry weight
Ji et al. (28)	2	5	5	−1.74	−3.32, −0.16	Tissue wet-dry weight
Ji et al. (28)	2	5	5	−1.48	−2.98, 0.01	Tissue wet-dry weight
Song et al. (24)	2	6	6	−0.42	−1.57, 0.73	Tissue wet-dry weight
Duan et al. (50)*	2	14	14	−57.12	−73.28, −40.97	Histology
**Summary statistic**	**Median** **=** **2**	**Σ** **=** **232**	**Σ** **=** **210**	${\bar{\text{X}}}_{\text{Weighted}}$ **=** **−23.67**	${\bar{\text{X}}}_{\text{Weighted}}$ **=** **−28.12**, **−19.21**	

Study marked (*) was excluded because it is an extreme outlier with influential statistical leverage.

## Discussion

Our meta-analysis showed that IA-SCI is beneficial when administered at the time of reperfusion in rat MCAO models. However, there are several considerations that must be made when interpreting these results. For example, the meta-analyses for our primary endpoints yielded significant and substantial heterogeneity, likely owing to a number of differing study characteristics (e.g., inter-lab variability, MCAO duration, saline infusion parameters). In contrast, aside from the MCAO and treatment parameters, experimental models appear to be relatively homogenous, as young and healthy rats are the most common model employed to study the efficacy of IA-SCI (with concurrent control groups). On average, the duration of MCAO was 2 h (range: 1 h–permanent MCAO). In all studies, the MCAO duration resulted in ≥ 30% hemispheric infarction in all control animals (range 30–64% hemispheric infarction), and this injury size is proportionally comparable to malignant infarction seen clinically (64). Thus, these models have face validity in that they represent severe stroke cases, including patients that benefit little from reperfusion therapy alone. Our trim-and-fill analyses revealed that our primary endpoints may be subjected to publication bias, as indicated by few null results in the published literature. During our analysis, we noticed a paucity of safety studies regarding IA-SCI, and few authors clearly justified the model choice and dosing parameters used in their study. Altogether, IA-SCI has demonstrable efficacy in animal models of MCAO, but there are limitations of the current literature that carry significant implications.

Our methodological quality analysis revealed that more studies are required to better predict the efficacy of IA-SCI treatment in clinical settings. We developed our scale in alignment with STAIR and RIGOR guidelines (41, 42), with the understanding that IA-SCI is relatively novel. Therefore, particular translational elements (i.e., age, sex) may not have been extensively explored. Accordingly, no studies investigated whether IA-SCI had similar efficacy in females compared to males, or aged compared to young animals. These results are pertinent, as multiple studies have shown that treatment efficacy may be reduced when experimentally considering age and comorbidities (34, 65). Indeed, age and certain comorbidities (e.g., hypertension) predict both stroke incidence and outcome, and thus future studies with these conditions are critical in understanding the impact of this treatment in its primary demographic. Moreover, as females are underrepresented in preclinical and clinical stroke research, future studies on this issue are critical (41, 42, 66). Finally, because neuroprotective efficacy has mostly been shown in the current literature using short-term (median = 48 h) assessments, future studies should consider comprehensive, long-term outcome assessments in order to complete our understanding of the neuroprotective efficacy of IA-SCI [e.g., shed light on persistence of neuroprotection, residual deficits; (42, 67)]. In sum, our analysis of methodological quality revealed a few opportunities for future study that are essential to understanding the parameters under which IA-SCI is efficacious. Because multiple studies have shown that treatment efficacy decreases in translationally-rigorous research, one might predict that IA-SCI may be less effective than the current literature suggests (34, 37, 65).

Importantly, few studies stated the assumptions used in determining their “dose” of infused saline. In our previous study, we assumed that humans could tolerate no more than 1 L of saline, infused at a rate of ~30 mL/min (68). Our initial calculations revealed an approximate range of saline infusion volumes that may be clinically applicable: 1.4 mL (scaled to brain weight), 4 mL (scaled to blood volume) and 4.4 mL (scaled to body mass). The results of our calculations were similar to those obtained by Ding et al. (23), however their group suggested that perhaps less saline (500–750 mL) could be given to patients at a faster rate (50–70 mL/min) (23). Our calculated infusion volumes are almost 3-fold greater than what has been used clinically with this method [e.g., in (21, 69) where ~350 mL of saline was infused]. Scaling to what has been done clinically so far, we calculate an IA-SCI volume of between 0.5 mL (scaled to brain weight) and 1.4 mL (scaled to body mass), which is notably lower than all of the animal studies reviewed here. In this meta-analysis, the average infusion volume across studies was 6 mL. Accordingly, animal studies may be overestimating benefit. Other parameters of the infusion procedure are critical to consider while modeling this treatment approach. For example, 17 of the studies (~89%) infused saline that was 20°C or cooler (median: 10°C; range: 0–23°C). IA-SCI in animal models involves direct catheterization of the external carotid artery (near the carotid bifurcation), whereas in patients, endovascular therapies are performed *via* femoral catheterization and the catheter is advanced to the injured brain region. Thus, in patient studies, the saline within the catheter is warmed by surrounding blood [minimized as catheter insulation is improved, e.g., (70)]. Indeed, a phantom model constructed by Choi et al. (69) showed that saline infused by a femoral catheter, running to the “internal carotid artery,” warmed to ~25°C at the catheter tip. Such warming is less likely to occur in animal studies of IA-SCI (69, 71). These results are important for investigators to consider given the current methodology of ischemic stroke treatment and limitations with current medical equipment and experimental modeling.

Interestingly, our meta-regression models were relatively ineffective at identifying factors that are known to contribute to the efficacy of TH (e.g., hypothermic depth and duration driven by saline temperature, volume, and rate). Of note, we expected our meta-regression model to identify MCAO duration as a contributor to treatment efficacy, as preclinical and clinical studies have repeatedly identified this to be an important modifier of efficacy (72–74). In line with our expectations, only one study in our data set investigated treatment efficacy in the context of multiple MCAO durations, and found waning treatment efficacy as MCAO durations increased, with no effect of this treatment following a 2.5 and 3 h MCAO duration (60). We expect that our meta-regression models were not successful (i.e., are inconsistent with current literature) for a few reasons. First, meta-regression models do not have sufficient statistical power if the number of studies are relatively low (75). Indeed, the number of studies that assessed edema were so low that we could not analyze the full meta-regression model, due to limited degrees of freedom. Second, meta-regression power is undermined when between study variance is large (i.e., τ^2^-values are high). In our study, between-study variation was common, as the model for our primary endpoints returned significant heterogeneity statistics. Third, our analysis may also suggest that the actual cooling effect of IA-SCI is less important than other mechanisms or a ceiling effect has been reached. However, at this time, there is not enough data to discern the key treatment parameters from the existing literature.

Pre-clinical research is well-suited for studying adverse events associated with disease, and thorough investigation of these events provide critical clinical information. During our analysis, we noticed that 58% of studies mentioned exclusions for a variety of reasons. Of particular interest were exclusions related to adverse bleeding events. Theoretically, TH may induce coagulopathy, highlighting the need for future studies to emphasize this complication (76, 77). Often, bleeding events were attributed to vascular perforation by the occluding device used in the MCAO model. To our knowledge, no studies have included bleeding complications related to chilled saline treatment as a primary endpoint in models of MCAO. Our previous study in ICH investigated the safety and efficacy of IA-SCI in the collagenase model of ICH, where bleeding evolves over hours (68). We found that IA-SCI did not appear to worsen bleeding when administered shortly (~30–60 min) after collagenase infusion, and did not improve long-term (day 28 post-ICH) behavioral or histological outcomes. However, our treatment was relatively mild compared to some studies included in this meta-analysis, because it was scaled to an infusion volume that would be clinically tolerable in patients [3 mL of room temperature (22°C) saline infused over 20 min in rat; (68)]. Although collagenase-induced hemorrhage shares some pathophysiological overlap with hemorrhagic transformation in ischemic stroke (e.g., blood-brain barrier breakdown *via* matrix metalloproteinases), future studies in focal ischemia with clinically-relevant elements (e.g., age, hypertension, coagulopathy) will be important to more definitively establish the safety of this treatment.

The only clinically-established neuroprotective interventions for focal cerebral ischemia are endovascular therapies [i.e., tPA administration, and/or MT; (4)]. To our knowledge, only one study has administered chilled saline in tandem with endovascular therapies, and that was in a non-human primate embolic MCAO model (78). However, this study did not meet our *a priori* inclusion criteria because it only investigated the combined effects of IA-SCI + tPA vs. tPA, without a sham IA-SCI or IA-SCI only treatment group. Thus, this study would be difficult to combine with the studies included in our meta-analysis. The authors thoroughly investigated the incremental benefit of IA-SCI with respect to reperfusion status and concluded that IA-SCI appears to provide acute histological benefit in animals with complete and partial reperfusion, though to a lesser degree in animals with partial reperfusion. In animals with no reperfusion, IA-SCI was unsuccessful at providing neuroprotection, and mortality was high in both groups. Animals that received IA-SCI treatment had better behavioral outcomes following a long-term (30-day) survival (main effect), but histological benefit could not be shown at this time. Given the ethical complexity and cost in the use of non-human primates, the typical control groups used in IA-SCI literature would be difficult to justify, but would be necessary in understanding the individual mechanistic contributions of IA-SCI alone and potential interactions with endovascular therapies. Thus, future rodent studies should consider a factorial design to investigate whether IA-SCI enhances behavioral and histological outcomes when administered alongside endovascular therapy and these studies will be foundational, as IA-SCI will likely be administered as an adjunct therapy to endovascular therapies, rather than a stand-alone treatment. Additionally, the safety and efficacy of this combinatorial treatment will be critical to establish, as tPA is known to increase hemorrhage risk, which may interact with IA-SCI (79, 80). Moreover, because tPA is a thrombolytic enzyme, hypothermia methods such as IA-SCI may hinder the effectiveness of tPA through kinetic inhibition (81). Very few humans have received IA-SCI so our understanding of its safety profile is relatively poor (26, 69). Although these early studies showed that IA-SCI appears to be safe and feasible, future preclinical safety and efficacy studies may shed light on possible contraindications and eligibility with this treatment.

### Limitations and Future Directions

One major question that remains following this meta-analysis is whether the depth and duration of TH offered by IA-SCI is sufficient to promote neuroprotection following severe ischemic insults (82). Indeed, MCAO duration is a well-known modifier of treatment efficacy, and often more aggressive treatments (e.g., longer and deeper TH protocols) are required to achieve persistent neuroprotection following greater intervention delays (42, 83), though this area is controversial as others have claimed that brief hypothermia is similarly effective, highlighting the need for rigorous dose-response studies for optimal TH protocols (84). IA-SCI offers a relatively short TH duration (<1 h) that is limited by saline volumes that are physiologically tolerable. Currently, it is unknown whether supplemental TH methods may provide incremental benefit beyond that observed with IA-SCI, especially following severe insults [e.g., those in (60)]. Future studies may feature an IA-SCI protocol to rapidly induce cooling, and further prolong cooling by TH methods that last for hours or days [e.g., nasopharyngeal methods; (19)].

Our endpoint selection criteria was limited to three endpoints, two of which are recommended by published guidelines [i.e., the STAIR and RIGOR guidelines; (41, 42)]. Although we initially planned to investigate long-term behavioral and histological outcomes, we found a heavy reliance on short-term histological and behavioral analysis (i.e., median latest assessment time = 48 h). Future studies must consider chronic [minimum 1 month; (42)] behavioral and histological analysis, as long-term outcomes are critical for understanding the persistence of neuroprotection, possible residual deficits, and potential complications.

Our *a priori* study inclusion criteria were relatively strict, with the conditions that studies used concurrent control groups in their statistical comparisons, and that IA-SCI was compared to a sham IA-SCI control group. This resulted in the exclusion of a few noteworthy studies. First, as mentioned above, Wu et al. (78) conducted a study in non-human primates where tPA-treated animals were compared to tPA + IA-SCI animals, but because this design is relatively unique in the IA-SCI literature, without a sham IA-SCI infusion group and pure IA-SCI treatment group, the results are difficult to compare to existing studies. Caroff et al. (70) performed IA-SCI in canines, but due to a small sample size in their control group, historical controls were used to boost the sample size, ultimately leading to the conclusion that IA-SCI appears to provide benefit in treated animals compared to controls. This study represents one of the few IA-SCI studies that we are aware of that have been conducted in non-rodent species. Altogether however, due to the general paucity of non-rodent animal species used in IA-SCI studies, combining these studies *via* meta-analysis with their relatively unique methodologies [e.g., embolic stroke in (78), and novel insulated catheter in (70)] would likely add to the already great statistical heterogeneity we observed in our meta-analysis. Nonetheless, those studies certainly add to the growing weight of evidence supporting the use of IA-SCI in focal ischemia.

Our qualitative analysis suggested that study designs used to investigate the efficacy of chilled saline treatment are relatively homogenous. For example, only one study used female animals, but did not consider sex as a biological variable (49). No studies considered age or comorbidities in their analysis of IA-SCI efficacy on ischemic stroke outcomes. Recent studies have shown that effect sizes are severely reduced (and risk of complications are increased) when age and comorbid conditions are considered, emphasizing a key area for future investigations (34, 37).

Our study focused specifically on the use of chilled saline administered into the carotid artery at the time of reperfusion. Therefore, we did not consider intra-arterially delivered treatments such as magnesium sulfate, stem cells, or other therapies. However, these have been recently reviewed by Link et al. (85).

Meta-analysis is an ever-changing method, evolving as new studies are published. As such, our data should not be taken as definitive evidence to support or refute the use of IA-SCI in the context of cerebral ischemia. Of note, our analysis of publication bias (i.e., Egger regression and trim-and-fill methods) suggested that our meta-analysis lacked null data that may have been found in so-called “gray literature.” However, our inclusion criteria were limited to peer-reviewed publications and thus null results available in alternative formats were not actively sought out. The limited number of null results in the context of relatively small sample sizes does suggest a positive publication bias, and several authors stress the importance of publishing results that are null (and negative) in order to inform the evidence-base surrounding a treatment (86).

## Summary

Translational pre-clinical research is conducted with the goal of developing and testing therapies for patient use (40). Because current therapies for focal ischemia do not improve outcomes for all that receive them, we sought to quantify whether a potential co-therapy, IA-SCI, could reduce brain damage and functional disability in animal models of MCAO. Our results suggest that IA-SCI is effective at reducing brain damage and improving behavioral outcomes when administered at the time of reperfusion in MCAO. Currently, studies have been performed in homogenous models, providing an excellent foundation for future study. Thus, future studies should consider integrating heterogeneous experimental design elements and longer survival times to establish the scope and magnitude of benefits observed with IA-SCI to inform its use in clinic.

## Data Availability Statement

The original contributions generated in the study are included in the article/Supplementary Materials, further inquiries can be directed to the corresponding author.

## Author Contributions

LL and FC were involved in study conceptualization and project administration. LL and CD were involved in data curation and analysis. LL, CD, and FC wrote the original draft. LL, FC, CD, BF, and MA were involved in expert review and editing. All authors contributed to the article and approved the submitted version.

## Conflict of Interest

The authors declare that the research was conducted in the absence of any commercial or financial relationships that could be construed as a potential conflict of interest.
